# Comment on ‘SARS-CoV-2 suppresses anticoagulant and fibrinolytic gene expression in the lung’

**DOI:** 10.7554/eLife.74268

**Published:** 2022-01-11

**Authors:** Ethan S FitzGerald, Amanda M Jamieson

**Affiliations:** 1 Division of Biology and Medicine, Department of Molecular Microbiology and Immunology, Brown University Providence United States; Kobe Pharmaceutical University Japan; Radboud University Medical Centre Netherlands

**Keywords:** COVID-19, coagulation, SARS-CoV-2, Human

## Abstract

Mast et al. analyzed transcriptome data derived from RNA-sequencing (RNA-seq) of COVID-19 patient bronchoalveolar lavage fluid (BALF) samples, as compared to BALF RNA-seq samples from a study investigating microbiome and inflammatory interactions in obese and asthmatic adults (Mast et al., 2021). Based on their analysis of these data, Mast et al. concluded that mRNA expression of key regulators of the extrinsic coagulation cascade and fibrinolysis were significantly reduced in COVID-19 patients. Notably, they reported that the expression of the extrinsic coagulation cascade master regulator Tissue Factor (F3) remained unchanged, while there was an 8-fold upregulation of its cognate inhibitor Tissue Factor Pathway Inhibitor (TFPI). From this they conclude that “pulmonary fibrin deposition does not stem from enhanced local [tissue factor] production and that counterintuitively, COVID-19 may dampen [tissue factor]-dependent mechanisms in the lungs”. They also reported decreased Activated Protein C (aPC) mediated anticoagulant activity and major increases in fibrinogen expression and other key regulators of clot formation. Many of these results are contradictory to findings in most of the field, particularly the findings regarding extrinsic coagulation cascade mediated coagulopathies. Here, we present a complete re-analysis of the data sets analyzed by Mast et al. This re-analysis demonstrates that the two data sets utilized were not comparable between one another, and that the COVID-19 sample set was not suitable for the transcriptomic analysis Mast et al. performed. We also identified other significant flaws in the design of their retrospective analysis, such as poor-quality control and filtering standards. Given the issues with the datasets and analysis, their conclusions are not supported.

## Introduction

Since the emergence of SARS-CoV-2 in December of 2019, there have been over 230 million reported cases and more than 4.7 million deaths (Dong et al., 2020). The scientific community has worked to rapidly advance our molecular and clinical understanding of COVID-19 (the disease caused by SARS-CoV-2) pathogenesis to develop lifesaving interventions. While the integration of diverse fields into the effort to understand this emergent disease can augment approaches, the rush of many disparate research teams to contribute to the infectious disease field at this time also holds significant risks. Given the importance and clinical relevance of COVID-19 research findings, including the retrospective examination of publicly available datasets, it is essential that the published data adheres to rigorous standards of quality control and certainty. In Mast et al, the authors used datasets from two published studies that performed bulk RNA-sequencing of bronchoalveolar lavage cells to specifically look at changes in the coagulation cascade (Mast et al., 2021). Samples from Zhou et al. contained the COVID-19 patient data and samples from Michalovich at el. served as the control group. In Zhou et al., to identify the etiological agent of COVID-19, total RNA content derived from the BALF of 9 human patients from the initial outbreak in China’s Wuhan Province was sequenced (Zhou et al., 2020). Michalovich et al. analyzed transcriptomic RNA sequencing libraries to understand how obesity, asthma, and smoking status amplified the dysbiosis of microbiome and immune interactions (Michalovich et al., 2019).

In Mast et al. the authors concluded that the extrinsic coagulation cascade regulation in the lung was not majorly impacted by SARS-CoV-2. They proposed that the bradykinin storm mediated pathogenesis originally proposed in Garvin et al., 2020 drove coagulopathies along with suppressed fibrinolysis. However, our reanalysis of the data-sets and the experimental design utilized by Mast et al. revealed the following serious issues that bring into question these conclusions; (1) the control group from Michalovich et al. contains mostly samples that are not from healthy lungs and many samples are from people with multiple comorbidities, (2) the two groups that are compared use fundamentally dissimilar library preparation methods that cannot be validly compared, and (3) Zhou et al. has insufficient read depth for it to be used for differential expression analysis. These issues are not readily observable in the published text of Mast et al., due to the use of Log-2 fold change and fold change in the text and figures, as well as the inclusion of only counts per million normalized counts in the supplemental files. This method of data reporting obscures the extremely low counts for many genes of interest. Many other publications in the field, including bioinformatics analyses, in-vitro studies, clinical research, and post-clinical autopsies directly contradict the findings of Mast et al (Subrahmanian et al., 2021; Rosell et al., 2021; FitzGerald et al., 2021). The initial publication of inaccurate findings could have been avoided by applying quality control standards to the libraries included in the analysis. These issues and clear contradictory evidence in the field, seriously compromise the accuracy of the differential expression analysis in Mast et al., and the validity of the conclusions reached by the authors.

## Results

### The designation of Michalovich et al. as a “Healthy Control” for differential expression analysis

**Figure 1.**
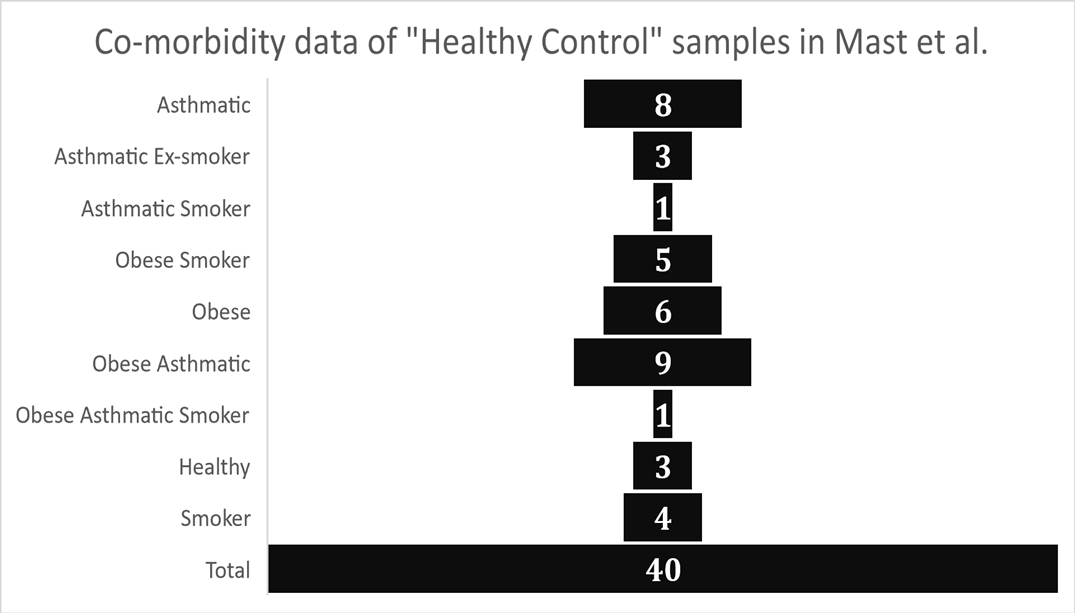
Graphical representation of health meta-data from control group samples as reported in Garvin et al. (Supplementary file 1 – “Patient Meta-data”).

The first issue we identified was related to the designation of the BALF bulk RNA-sequencing samples from Michalovich et al (GEO data set - PRJNA434133) as “Healthy Controls” by Mast et al. Analysis of the meta-data associated with the described “Healthy Control” subjects published in Michalovich et al. demonstrates that their samples were overall not healthy and also not representative of the average American population in terms of obesity (CDC, 2021b) (42.4%), smoking rates (CDC, 2020) (14.0%), and asthma (CDC, 2021a) (8%). Metadata reported from Michalovich et al. (also reported in the supplement of Garvin et al.) indicates that only 3 samples out of 40 had were reported as non-asthma, non-smoking, and non-obese (7.5%). The “healthy controls” were reported as 52.5% obese, 27.5% active smokers, and 55% asthmatic, with many individuals having multiple of these co-morbidities. (Figure 1).

The issues with using the samples from Michalovich et al. as the healthy control samples is made clear in the findings of the original manuscript. The study found significant changes in systemic and pulmonary inflammation when comparing individuals with obesity, asthma, or smoking history to their healthy subjects. Specifically, they found elevated levels of circulating inflammatory mediators (IL-6, IL-2, TNF-α, etc.) and proteins regulating coagulation (C-reactive protein, Fibrinogen, Serum Amyloid Alpha). Additionally, they reported significant changes in BALF concentrations of C reactive protein, Serum Amyloid Alpha, IL-5, and other proteins that would impact coagulation and inflammation. Gene ontology analysis of transcriptional differences in the BALF that were published in Michalovich et al. identified significant enrichments in tissue remodeling and inflammation ontologies amongst obese and asthmatic groups relative to the three healthy controls. These significant inflammatory, pro-coagulant, and transcriptional changes within the samples that Mast et al. designated as “healthy controls” have many overlapping similarities with the phenotypic changes that are associated with SARS-CoV-2. Such changes would significantly disrupt the ability to accurately characterize SARS-CoV-2 differential expression as these comorbidities are not controlled for in Mast et al. The presence of such disparate sample types within the “healthy control” group does not yield an averaged and more representative control group as was implied in Mast et al., instead the pro-coagulant transcriptional changes associated with the co-morbidities observed in much of the control group would likely mask relevant COVID-19 induced transcriptional changes. Additionally, averaging of highly disparate samples within the control group during differential expression analysis would not yield a more representative data set, but rather would generate a noisy control group with averages significantly weighted towards more abundant sample types.

### Dissimilar library preparation methods of Michalovich et al. (transcriptomic) and Zhou et al. (total RNA) are not comparable

Based on the description of the RNA sequencing library preparation methods described in Zhou et al. and Michalovich et al., very different approaches were used to prepare sequencing libraries. The type of library preparation can significantly modify the RNA content of sequencing libraries via polyA enrichment, rRNA depletion, and other major differences in molecular processes underlying library preparation. Dissimilar libraries, particularly those with non-similar polyA enrichment and ribosomal RNA (rRNA) depletion, cannot reliably be used for differential expression analysis with transcripts per million (TPM) based normalization, which Mast et al. utilized in their analysis (Zhao et al., 2020).

Michalovich et al. uses libraries that are enriched for mRNA via polyA enrichment, while Zhou et al. does not. Michalovich et al. also uses a TruSeq Stranded RNA library Prep kit with RiboZeroTMGold ribodepletion probes. This library preparation approach yields libraries that are selectively depleted of ribosomal reads (which are the predominant RNA species in cells), while enriching for mature mRNA transcripts (Lodish et al., 1999). By contrast, Zhou et al. used HighPure Viral RNA preparation kits to purify RNA for sequencing and did not describe any enrichments or depletion during library preparation. Given that the purpose of the sequencing analysis performed by Zhou et al. was to identify the viral etiological agent of COVID-19, polyA enrichment was likely not performed to avoid depleting viral transcripts that are not poly-adenylated. This library preparation approach yields libraries that contain total RNA content from host cells, including viral RNAs, rRNAs, and all forms of eukaryotic transcripts such as pre-mRNA, ncRNAs, mRNAs, and other rare RNAs.

**Figure 2.**
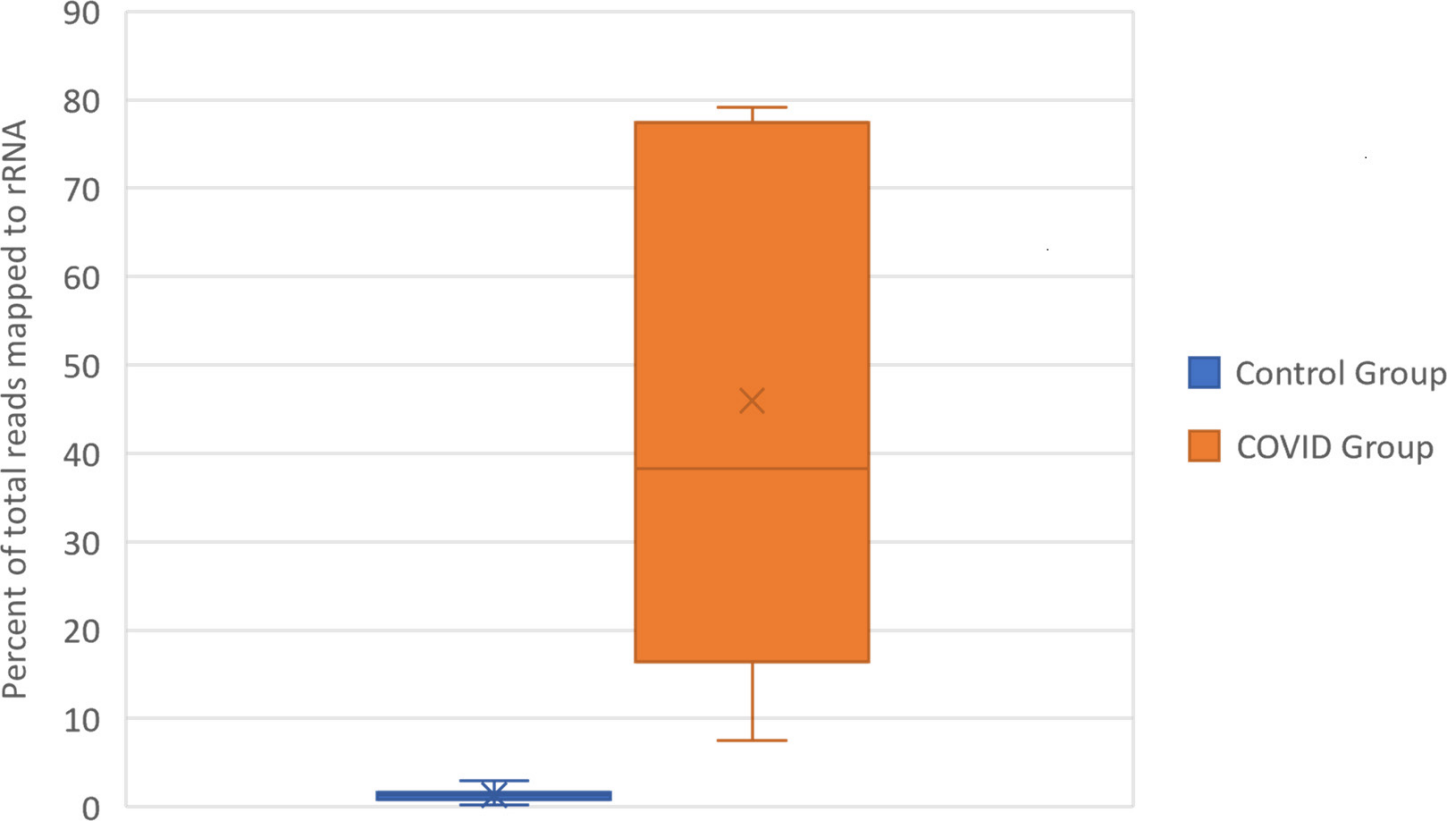
Box and Whisker plot of the percent of rRNA reads. Control group and SARS-CoV-2+ fastq files were accessed from public repositories and aligned with CLC genomics workbench as described in Garvin et al. Count data for known RefSeq rRNA features were sorted from CLC generated count tables and summed per sample. rRNA count sums were then divided by the total counts per sample to generate percentages.

To confirm the functional differences in library preparation methods, we analyzed the proportion of reads aligning to rRNA transcripts using the same CLC genomics alignment settings and reference transcriptomes described in Mast et al. This confirmed that the total rRNA content was at a significantly greater proportion in SARS-CoV-2+ patient samples relative to control samples. (Figure 2, Supplementary file 1) The amount of rRNA reads in the SARS-CoV-2+ patient samples would also significantly decrease the mRNA transcriptome coverage of the SARS-CoV-2+ libraries. These discrepancies in RNA composition between libraries generated by Michalovich et al. and Zhou et al. would unacceptably distort TPM based count normalization and library size normalization during differential expression analysis, both of which rely on the assumption that the libraries contain the same kinds of RNAs (Evans et al., 2018).

### Insufficient read depth of samples from Zhou et al.

RNA-seq approaches for differential expression analysis require that enough sequencing reads be collected to accurately quantify the total expression of transcripts across the genome. In order to have statistical meaningful numbers of reads mapping to each gene for differential expression analysis minimum read depth requirements must be met. If a particular transcript is lowly expressed relative to other transcripts, then a low number of reads may be stochastically detected during sequencing. Such a dynamic could artificially inflate or deflate the relative expression of a particular transcript, especially when normalization approaches are applied to compare libraries sequenced at different depths or with radically different RNA compositions. It is generally accepted in the field that experiments investigating eukaryotic global gene expression typically require at least 30 million poly-A and ribo-depleted reads per sample (Williams et al., 2014). In human cells, such as those investigated by Mast et al. approximately 80% of transcripts expressed at >10 fragments per kilobase per million mapped reads (FPKM) are accurately quantifiable with about 36 million 100 bp paired end reads (Sims et al., 2014).

**Figure 3.**
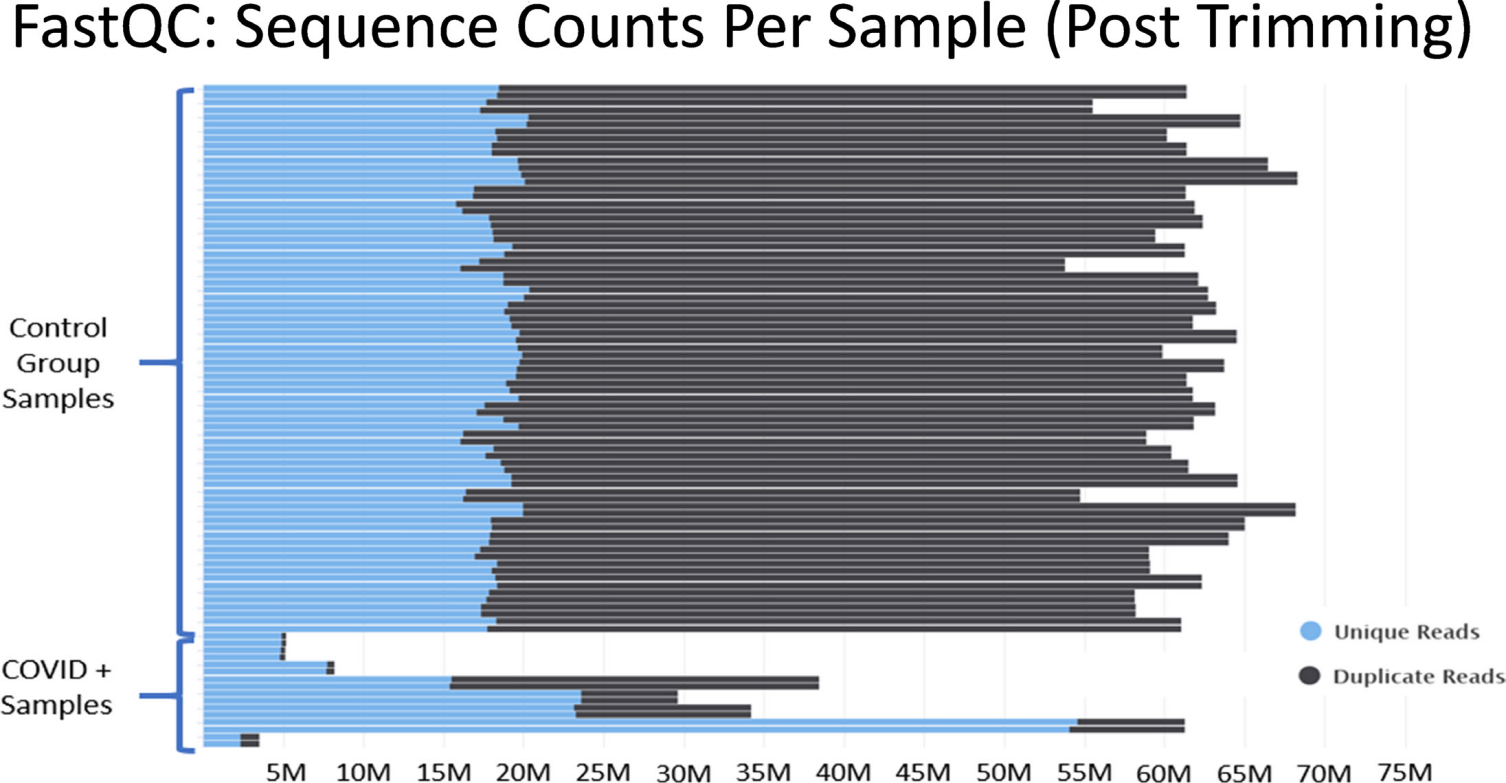
Histograms of the total number of reads per sample after adapter and quality trimming was performed with TrimGalore. This graphic was generated by analyzing trimmed fastq files using the package FastQC, and then processing the FastQC output into the package MultiQC. MultiQC was used to generate the histogram.

In Mast et al., there are major discrepancies in the relative depths of the sequencing libraries used for the “healthy control” samples and the SARS-CoV-2+ patient samples (Figure 3). Of the nine SARS-CoV-2+ samples from Zhou et al., four contain less than 10 million reads total, an additional four contain between 30 million to 40 million reads, and one sample contains 60 million reads. As described in Figure 2, many of the samples from Zhou et al. contain high proportions of ribosomal RNA reads, which would further compromise coverage of the mRNA transcriptome in the COVID-19+ sample set. These significant issues are most clearly substantiated by the observation that many essential genes of interest reported in Table 1 of Mast et al. have very few or even no mapped reads in COVID-19 patient samples. (Supplementary file 2 and Source data 2) This makes fold change values and differential comparisons, particularly after normalization, unreliable and not representative of the actual biological RNA content or transcriptional activity.

To illustrate a specific instance, Mast et al. report that thrombomodulin (THBD) expression in the BALF was decreased by 2200% during SARS-CoV-2 infection. They reported the expression level to be approximately 9.6 counts per million reads in COVID-19 infection and 224 counts per million reads in the control sample set. However, at the level of raw counts, control samples averaged 8,377.68 counts while COVID-19 positive samples averaged 59.88 counts. The normalization process for the counts per million based normalization was further biased by the inclusion of between 16%–80% of the total rRNA in only COVID-19 samples. These rRNA reads would be included in the total number of mapped reads used to calculate the CPM normalization factor in a manner that was not consistent with the normalization of control samples. (CPM Normalization factor = [Total number of reads x 10^6]/[Total number of mapped reads]) Additionally, such a bias would significantly decrease the likelihood of detecting mRNA transcripts in the COVID + genes, including thrombomodulin transcripts. These confounding factors could bring into question the accuracy of the reported magnitude of the differential expression, the reported directionality of the differential expression, and the subsequent pathway analysis performed.

## Discussion

The significant issues we have identified regarding the heterogeneity of control samples, dissimilar library preparation methods, and insufficient sequencing depth collectively bring into doubt the validity of many of the conclusions drawn in Mast et al. The normalized manner in which the gene expression data were reported in the supplement and manuscript of Mast et al. made it difficult for reviewers and readers to identify these issues when analyzing the manuscript. Mast et al. additionally did not provide supplemental data regarding the raw reads that were processed during alignment, the raw counts that were normalized and processed during differential expression analysis, or any NGS quality control standards that should have been conducted by the authors before analyzing the data set. From our analysis of their raw data, we conclude that the sample set and experimental design implemented in Mast et al. are fundamentally flawed. The concerns are significantly magnified knowing that others researching COVID-19 are citing these poorly substantiated results in publications (Francischetti et al., 2021) or integrating these findings into their experimental design and future plans.

Upon processing the raw data as described in our results section, serious issues with relative sequencing depth quickly became apparent. Review of the count data which we have summarized in Table 2 and the differential expression results for genes of interest reported in Table 1 of Mast et al. demonstrate the flawed nature of this analysis. Overall, 23 out of 35 genes of interest reported in Mast et al. average less than 10 mapped reads per gene but were still included in the analysis. (Supplementary file 2 and Source data 2) 8 of those genes had zero mapped reads reported. (Supplementary file 2 and Source data 2) The fold change magnitudes reported for these genes are almost certainly not reflective of the actual biological context.

By far the most notable result reported in Mast et al. is the reported observation that tissue factor, the key initiator of the extrinsic coagulation cascade, is not significantly impacted by SARS-CoV-2 infection. They reported no significant difference in expression levels and concluded that tissue factor biology was not a significant factor in the thrombotic complications of SARS-CoV-2 in the lung. They postulated that COVID-19 may dampen tissue factor dependent mechanisms in the lung. This analysis was confounded by the above-described issues including relative depth, rRNA differences between control and COVID sample sets, and normalization This statement is important as the field has also begun converging on tissue factor as a key player in the pathogenesis and coagulopathy complications of SARS-CoV-2 infection. For instance, patients with COVID-19 have been shown to have elevated levels of tissue factor laden microvesicles circulating in their blood, along with other markers of the extrinsic coagulation cascade (Rosell et al., 2021). Further, autopsy studies of COVID-19 patients have also found that tissue factor protein expression is approximately doubled in the lungs of patients that succumbed to COVID-19 (Subrahmanian et al., 2021). Tissue factor upregulation in the BALF of COVID-19 patients has also been observed at the RNA level using both single cell and bulk RNA-sequencing, and the observed increase correlated with severity (FitzGerald et al., 2021). These major discrepancies with the field and the ultimate inaccuracies of several conclusions advanced by Mast et al. demonstrate that the design of retrospective analyses implemented in Mast et al. are fundamentally flawed and should not be integrated into future research findings.

At the time the manuscript was submitted, several higher quality data sets were available and the authors of Mast et al. should have redone their analysis on sample sets that were collected with the intent of resolving transcriptomic signatures to accurately characterize the host response to SARS-CoV-2 (Xiong et al., 2020; Liao et al., 2020; Xu et al., 2020). Additionally, sufficient metadata, raw NGS data outputs, and quality control reports should have been provided to reviewers at the time of submission. The research community relies on a dependable body of shared knowledge with well designed and controlled studies so that future research can proceed in the correct direction.

## Data Availability

All data generated or analysed during this study are included in the manuscript and supporting files; Source data files have been provided.

## Additional files

Supplementary file 1. This file contains a subset of count data derived from Source data 2. The genes included in the subset were generated using Ensembl Biomart. We first selected human genes (GRCh38.p13) as our data set, before then filtering to only include genes annotated as rRNA encoding (Gene type = rRNA). All rRNA genes identified in the data set with any counts were included.

Supplementary file 2. This file contains a subset of count data derived from Source data 2. The subset of genes include all mentioned genes of interest analyzed in the Mast et al., 2021 publication. All genes of interest from Mast et al. were included regardless of count totals.

Transparent reporting form

Source data 1. CLC Alignment QC Reports. This file contains quality control reports generated during our reproduction of the Mast et al., 2021 data analysis in CLC Genomics Workbench. They demonstrate that we implemented identical analyses based on their description of their methods and provide useful summary information about the alignment process.

Source data 2. Raw Count Tables. This file contains raw count data for all samples. These counts were generated during the CLC Genomics workbench based alignment process, which was performed as described in the methods of Mast et al., 2021.

Source data 3. EdgeR_DEGs_Mast. This file contains the output from applying the Mast_EdgeR_script.R code on the raw counts provided in Source data 2.

Source code 1. Mast_EdgeR_script.R. This file contains an R script which was produced based on correspondence with the authors of Mast et al. It reproduced the implementation of EdgeR which they utilized for differential expression analysis of

## References

[bib1] CDC (2020). Current Cigarette Smoking Among Adults in the United States. Centers for Disease Control and Prevention.

[bib2] CDC (2021a). Most Recent National Asthma Data. https://www.cdc.gov/asthma/most_recent_national_asthma_data.htm.

[bib3] CDC (2021b). Obesity is a Common, Serious, and Costly Disease. Centers for Disease Control and Prevention.

[bib4] Dong E, Du H, Gardner L (2020). An interactive web-based dashboard to track COVID-19 in real time. The Lancet. Infectious Diseases.

[bib5] Evans C, Hardin J, Stoebel DM (2018). Selecting between-sample RNA-Seq normalization methods from the perspective of their assumptions. Briefings in Bioinformatics.

[bib6] FitzGerald ES, Chen Y, Fitzgerald KA, Jamieson AM (2021). Lung Epithelial Cell Transcriptional Regulation as a Factor in COVID-19-associated Coagulopathies. American Journal of Respiratory Cell and Molecular Biology.

[bib7] Francischetti IMB, Toomer K, Zhang Y, Jani J, Siddiqui Z, Brotman DJ, Hooper JE, Kickler TS (2021). Upregulation of pulmonary tissue factor, loss of thrombomodulin and immunothrombosis in SARS-CoV-2 infection. EClinicalMedicine.

[bib8] Garvin MR, Alvarez C, Miller JI, Prates ET, Walker AM, Amos BK, Mast AE, Justice A, Aronow B, Jacobson D (2020). A mechanistic model and therapeutic interventions for COVID-19 involving a RAS-mediated bradykinin storm. eLife.

[bib9] Liao M, Liu Y, Yuan J, Wen Y, Xu G, Zhao J, Cheng L, Li J, Wang X, Wang F, Liu L, Amit I, Zhang S, Zhang Z (2020). Single-cell landscape of bronchoalveolar immune cells in patients with COVID-19. Nature Medicine.

[bib10] Lodish HA, Berk A, Zipursky SL, Matsudaira P, Baltimore D, Darnell EJ (1999). Molecular Cell Biology.

[bib11] Mast AE, Wolberg AS, Gailani D, Garvin MR, Alvarez C, Miller JI, Aronow B, Jacobson D (2021). SARS-CoV-2 suppresses anticoagulant and fibrinolytic gene expression in the lung. eLife.

[bib12] Michalovich D, Rodriguez-Perez N, Smolinska S, Pirozynski M, Mayhew D, Uddin S, Van Horn S, Sokolowska M, Altunbulakli C, Eljaszewicz A, Pugin B, Barcik W, Kurnik-Lucka M, Saunders KA, Simpson KD, Schmid-Grendelmeier P, Ferstl R, Frei R, Sievi N, Kohler M, Gajdanowicz P, Graversen KB, Lindholm Bøgh K, Jutel M, Brown JR, Akdis CA, Hessel EM, O’Mahony L (2019). Obesity and disease severity magnify disturbed microbiome-immune interactions in asthma patients. Nature Communications.

[bib13] Rosell A, Havervall S, von Meijenfeldt F, Hisada Y, Aguilera K, Grover SP, Lisman T, Mackman N, Thålin C (2021). Patients With COVID-19 Have Elevated Levels of Circulating Extracellular Vesicle Tissue Factor Activity That Is Associated With Severity and Mortality-Brief Report. Arteriosclerosis, Thrombosis, and Vascular Biology.

[bib14] Sims D, Sudbery I, Ilott NE, Heger A, Ponting CP (2014). Sequencing depth and coverage: key considerations in genomic analyses. Nature Reviews Genetics.

[bib15] Subrahmanian S, Borczuk A, Salvatore S, Fung K-M, Merrill JT, Laurence J, Ahamed J (2021). Tissue factor upregulation is associated with SARS-CoV-2 in the lungs of COVID-19 patients. Journal of Thrombosis and Haemostasis.

[bib16] Williams AG, Thomas S, Wyman SK, Holloway AK (2014). RNA-seq Data: Challenges in and Recommendations for Experimental Design and Analysis. Current Protocols in Human Genetics.

[bib17] Xiong Y, Liu Y, Cao L, Wang D, Guo M, Jiang A, Guo D, Hu W, Yang J, Tang Z, Wu H, Lin Y, Zhang M, Zhang Q, Shi M, Liu Y, Zhou Y, Lan K, Chen Y (2020). Transcriptomic characteristics of bronchoalveolar lavage fluid and peripheral blood mononuclear cells in COVID-19 patients. Emerging Microbes & Infections.

[bib18] Xu G, Qi F, Li H, Yang Q, Wang H, Wang X, Liu X, Zhao J, Liao X, Liu Y, Liu L, Zhang S, Zhang Z (2020). The differential immune responses to COVID-19 in peripheral and lung revealed by single-cell RNA sequencing. Cell Discovery.

[bib19] Zhao S, Ye Z, Stanton R (2020). Misuse of RPKM or TPM normalization when comparing across samples and sequencing protocols. RNA.

[bib20] Zhou P, Yang XL, Wang XG, Hu B, Zhang L, Zhang W, Si HR, Zhu Y, Li B, Huang CL, Chen HD, Chen J, Luo Y, Guo H, Jiang RD, Liu MQ, Chen Y, Shen XR, Wang X, Zheng XS, Zhao K, Chen QJ, Deng F, Liu LL, Yan B, Zhan FX, Wang YY, Xiao GF, Shi ZL (2020). A pneumonia outbreak associated with a new coronavirus of probable bat origin. Nature.

