# Supplementary material for: Comment on ‘SARS-CoV-2 suppresses anticoagulant and fibrinolytic gene expression in the lung’
Source: eLife. 2022 Jan 11;11:e74268. doi: 10.7554/eLife.74268 (PMC8752089; doi:10.7554/eLife.74268)
Supplement: Source data 1. — This file contains quality control reports generated during our reproduction of the Mast et al., 2021 data analysis in CLC Genomics Workbench. They demonstrate that we implemented identical analyses based on their description of their methods and provide useful summary information about the alignment process. [file elife-74268-supp3.zip › Source Data 1 - CLC Alignment QC Reports/SRR11092063_1 (RNA-Seq report).pdf]

Generated by: ethan
Date: Sat May 22 02:59:13 EDT 2021

Table of contents

1. Selected input sequences 3
    1.1 Sequence reads 3
    1.2 Reference Sequences 3
2. References 3
3. Read quality control 3
    3.1 Strand specificity 3
    3.2 Adapter read-through 3
    3.3 Paired distance 4
4. Mapping statistics 4
    4.1 Paired reads 4
    4.2 Match specificity (paired) 5
5. Fragment statistics 5
    5.1 Fragment counting (total) 5
6. Transcript length coverage 6

1. Selected input sequences

1.1 Sequence reads

Name	Number of reads	Longest read	Paired
SRR11092063_1 (paired, trimmed pairs)	134,146,638	150	yes

For paired data, there are two reads in a pair.

1.2 Reference Sequences

References	Length	Genes
164,291	597,293,918	164,291

2. References

3. Read quality control

3.1 Strand specificity

Strand specific setting	Both
Forward % of reads mapped	47.18
Reverse % of reads mapped	52.82
Reads with known strand	10,529,288
Ignored reads (wrong strand)	0
Ignored reads % (wrong strand)	0.00

Strand specificity refers to whether a read is generated by sequencing an RNA fragment in the Forward (5' to 3') or Reverse (3' to 5') orientation. In a strand-specific protocol almost all reads are generated from a specific orientation. In other protocols a mix of both orientations is expected.

3.2 Adapter read-through

Estimated % containing read-through adapters	1.21

This value is estimated from the number of mapped paired-end reads where one read is longer than the sequenced RNA fragment.
A small percentage of read-through adapters is unlikely to affect the quantification of expression, but their presence may lead to false positive variant calls. To remove a read-through adapter, run the "Trim Reads" tool on the input reads, using either Automatic read-through adapter trimming or a Trim Adapter List configured to remove the adapter sequence from the 3' end.

3.3 Paired distance

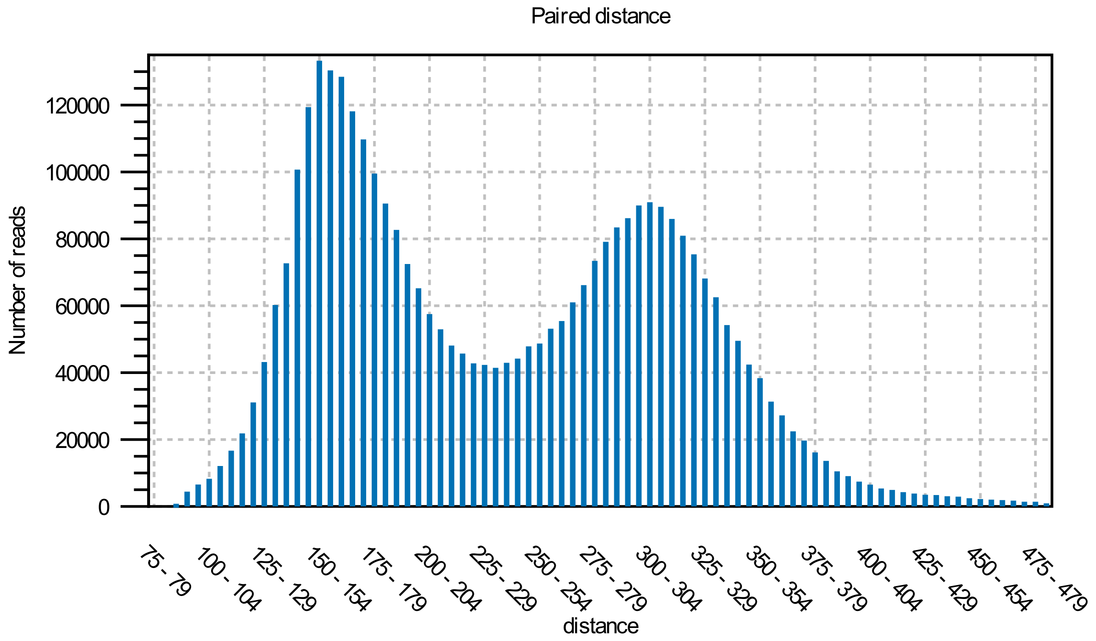

The graph shows the distribution of paired-end distances, which is equivalent to the distribution of sequenced RNA fragment sizes. There should be a single broad peak at the target fragment size. An asymmetric peak may indicate problems in size selection.

Reads	Paired distance estimates	Messages
SRR11092063_1 (paired, trimmed pairs)	75 to 483 bp	Few reads mapped as pairs so pair distance might not be accurate

4. Mapping statistics

4.1 Paired reads

	Number of sequences	%
Reads mapped in pairs	7,276,884	5.42
Reads mapped in broken pairs	3,252,404	2.42
Reads not mapped	123,617,350	92.15

	Number of sequences	%
Total	134,146,638	100.00

For paired data, there are two reads in a pair.

If a strand specific setting was used, 'Reads not mapped' will include those reads that were ignored because they mapped to the wrong strand.

Reads not mapped: >50% of the reads did not map. If the correct reference genome has been selected, this could indicate a serious problem with the sample.
• Check that the correct reference genome and any relevant gene/mRNA tracks have been provided.
• The mapping parameters may be too strict. Try resetting them to the default values.
• Try mapping the un-mapped reads against possible contaminants. If the sample is contaminated, enrich for the target species before library preparation in future experiments.
• Library preparation may have failed. Check the quality of the sample RNA.

4.2 Match specificity (paired)

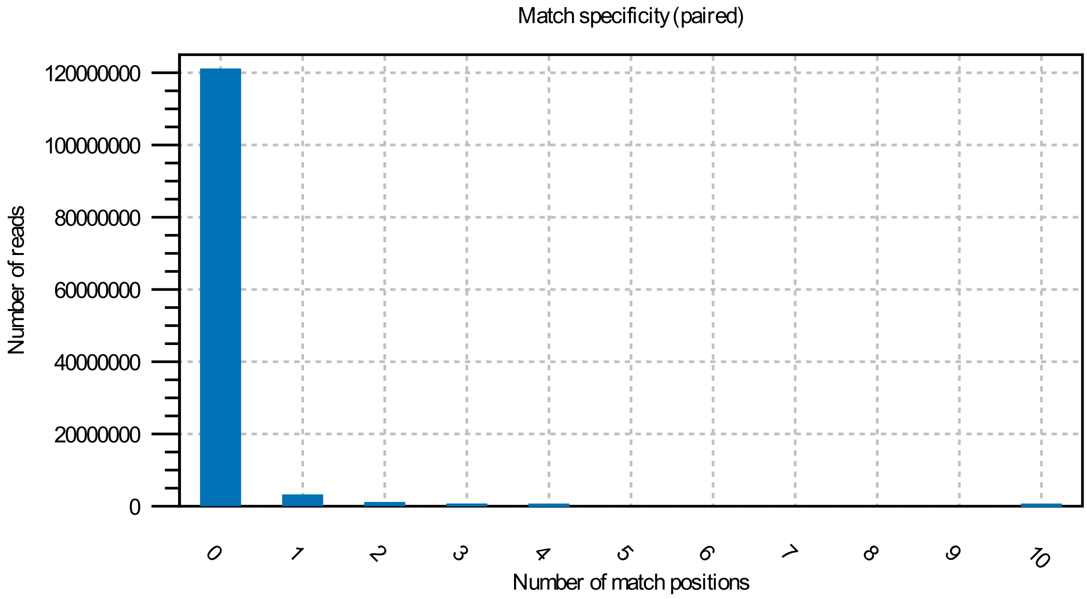

5. Fragment statistics

5.1 Fragment counting (total)

	Fragments	% of total
Counted fragments	3,638,442	5.42
- unique fragments	1,640,077	2.45
- non-specifically	1,998,365	2.98
Uncounted fragments	63,434,877	94.58

	Fragments	% of total
Total fragments	67,073,319	100.00

Default counting scheme ('Fragment counts'): An intact pair is counted as one, broken pairs are ignored.

6. Transcript length coverage

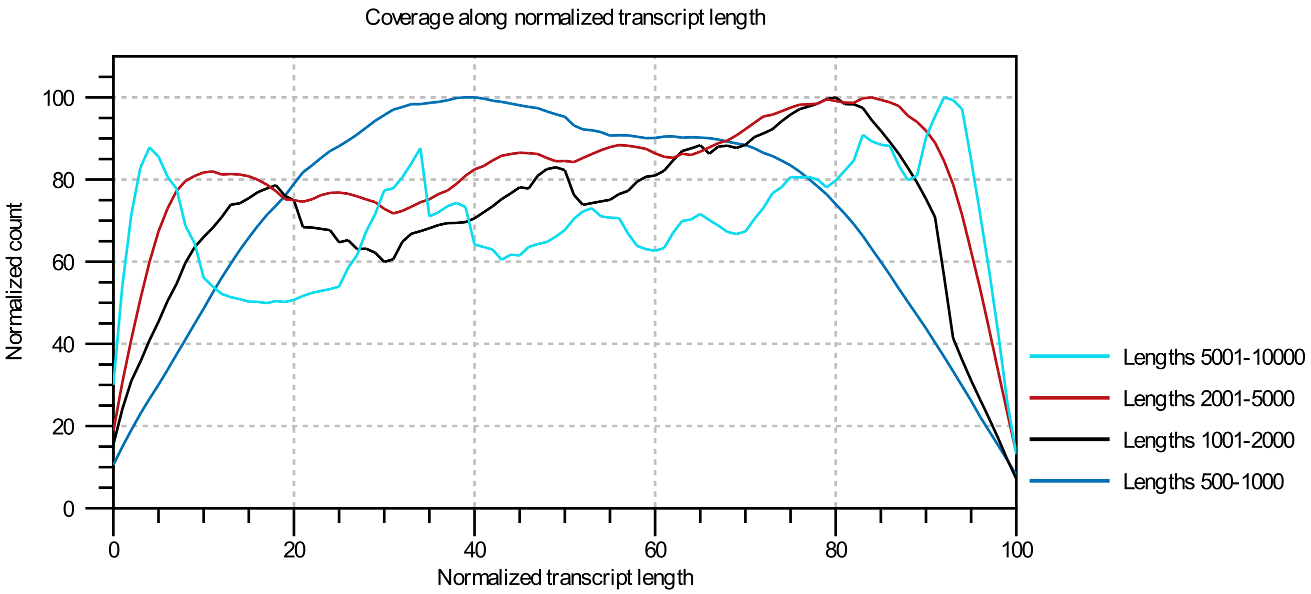

Expected coverage bias	Unbiased
Difference between average 3’ and 5’ normalized counts	9.60
% reads mapping to transcripts > 10000 bp	11.24
% reads mapping to transcripts 5001 - 10000 bp	28.90
% reads mapping to transcripts 2001 - 5000 bp	42.56
% reads mapping to transcripts 1001 - 2000 bp	14.91
% reads mapping to transcripts 500 - 1000 bp	2.23
% reads mapping to transcripts < 500 bp	0.16

The plot shows the normalized coverage across a transcript body for four different groupings of transcript length. The lines should be flat in the center of the plot, and the plot should be approximately symmetric. An erratic line may indicate that there are few transcripts in the given length range.
