# Supplementary material for: Comment on ‘SARS-CoV-2 suppresses anticoagulant and fibrinolytic gene expression in the lung’
Source: eLife. 2022 Jan 11;11:e74268. doi: 10.7554/eLife.74268 (PMC8752089; doi:10.7554/eLife.74268)
Supplement: Source data 1. — This file contains quality control reports generated during our reproduction of the Mast et al., 2021 data analysis in CLC Genomics Workbench. They demonstrate that we implemented identical analyses based on their description of their methods and provide useful summary information about the alignment process. [file elife-74268-supp3.zip › Source Data 1 - CLC Alignment QC Reports/SRR10571730_1 (RNA-Seq report).pdf]

Generated by: ethan
Date: Thu May 20 07:15:03 EDT 2021

## Table of contents

1. Selected input sequences ........ 3
    1.1 Sequence reads ........ 3
    1.2 Reference Sequences ........ 3
2. References ........ 3
3. Read quality control ........ 3
    3.1 Strand specificity ........ 3
    3.2 Adapter read-through ........ 4
    3.3 Paired distance ........ 4
4. Mapping statistics ........ 4
    4.1 Paired reads ........ 5
    4.2 Match specificity (paired) ........ 5
5. Fragment statistics ........ 5
    5.1 Fragment counting (total) ........ 5
6. Transcript length coverage ........ 6

# 1. Selected input sequences

## 1.1 Sequence reads

| Name | Number of reads | Longest read | Paired |
|---|---|---|---|
| SRR10571730_1 (paired, trimmed pairs) | 122,812,008 | 51 | yes |

For paired data, there are two reads in a pair.

## 1.2 Reference Sequences

| References | Length | Genes |
|---|---|---|
| 164,291 | 597,293,918 | 164,291 |

# 2. References

# 3. Read quality control

## 3.1 Strand specificity

| | |
|---|---|
| Strand specific setting | Both |
| Forward % of reads mapped | 0.92 |
| Reverse % of reads mapped | 99.08 |
| Reads with known strand | 90,163,475 |
| Ignored reads (wrong strand) | 0 |
| Ignored reads % (wrong strand) | 0.00 |

Strand specificity refers to whether a read is generated by sequencing an RNA fragment in the Forward (5' to 3') or Reverse (3' to 5') orientation. In a strand-specific protocol almost all reads are generated from a specific orientation. In other protocols a mix of both orientations is expected.

Strand specific setting: >90% of reads were mapped in the same orientation. Consider re-running the tool with a strand specific setting ("Forward"/"Reverse").

## 3.2 Adapter read-through

| | |
|---|---|
| Estimated % containing read-through adapters | 0.00 |

This value is estimated from the number of mapped paired-end reads where one read is longer than the sequenced RNA fragment.
A small percentage of read-through adapters is unlikely to affect the quantification of expression, but their presence may lead to false positive variant calls. To remove a read-through adapter, run the "Trim Reads" tool on the input reads, using either Automatic read-through adapter trimming or a Trim Adapter List configured to remove the adapter sequence from the 3' end.

## 3.3 Paired distance

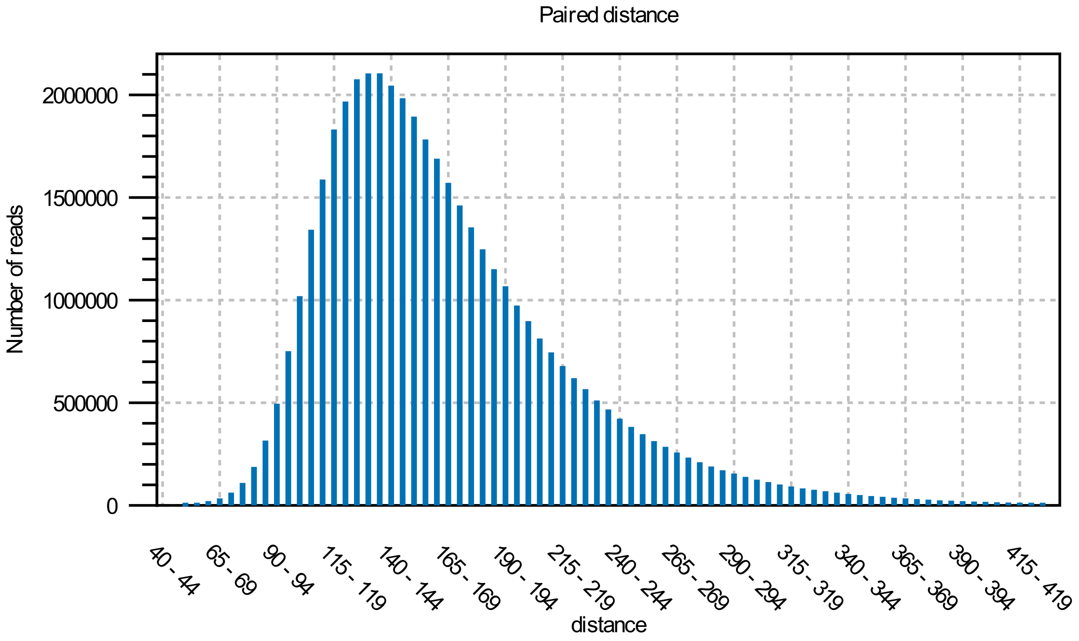


The graph shows the distribution of paired-end distances, which is equivalent to the distribution of sequenced RNA fragment sizes. There should be a single broad peak at the target fragment size. An asymmetric peak may indicate problems in size selection.

| Reads | Paired distance estimates | Messages |
|---|---|---|
| SRR10571730_1 (paired, trimmed pairs) | 43 to 431 bp | OK |

# 4. Mapping statistics

## 4.1 Paired reads

| | Number of sequences | % |
|---|---|---|
| Reads mapped in pairs | 87,772,816 | 71.47 |
| Reads mapped in broken pairs | 2,390,659 | 1.95 |
| Reads not mapped | 32,648,533 | 26.58 |
| Total | 122,812,008 | 100.00 |

For paired data, there are two reads in a pair.

If a strand specific setting was used, 'Reads not mapped' will include those reads that were ignored because they mapped to the wrong strand.

## 4.2 Match specificity (paired)

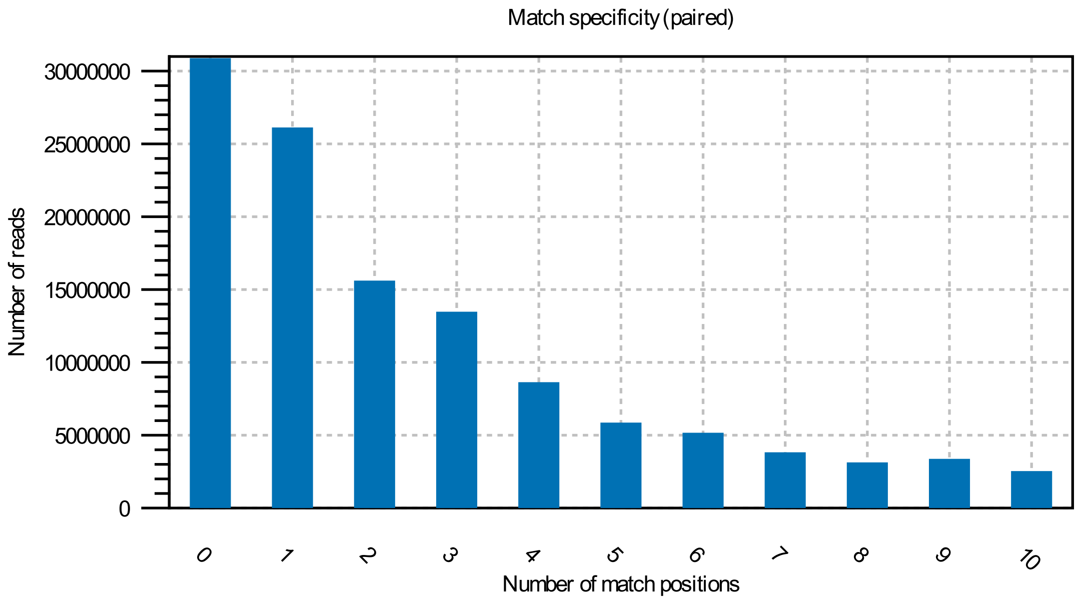


# 5. Fragment statistics

## 5.1 Fragment counting (total)

| | Fragments | % of total |
|---|---|---|
| Counted fragments | 43,886,408 | 71.47 |
| - unique fragments | 13,064,405 | 21.28 |
| - non-specifically | 30,822,003 | 50.19 |
| Uncounted fragments | 17,519,596 | 28.53 |

| | Fragments | % of total |
|---|---|---|
| Total fragments | 61,406,004 | 100.00 |

Default counting scheme ('Fragment counts'): An intact pair is counted as one, broken pairs are ignored.

# 6. Transcript length coverage

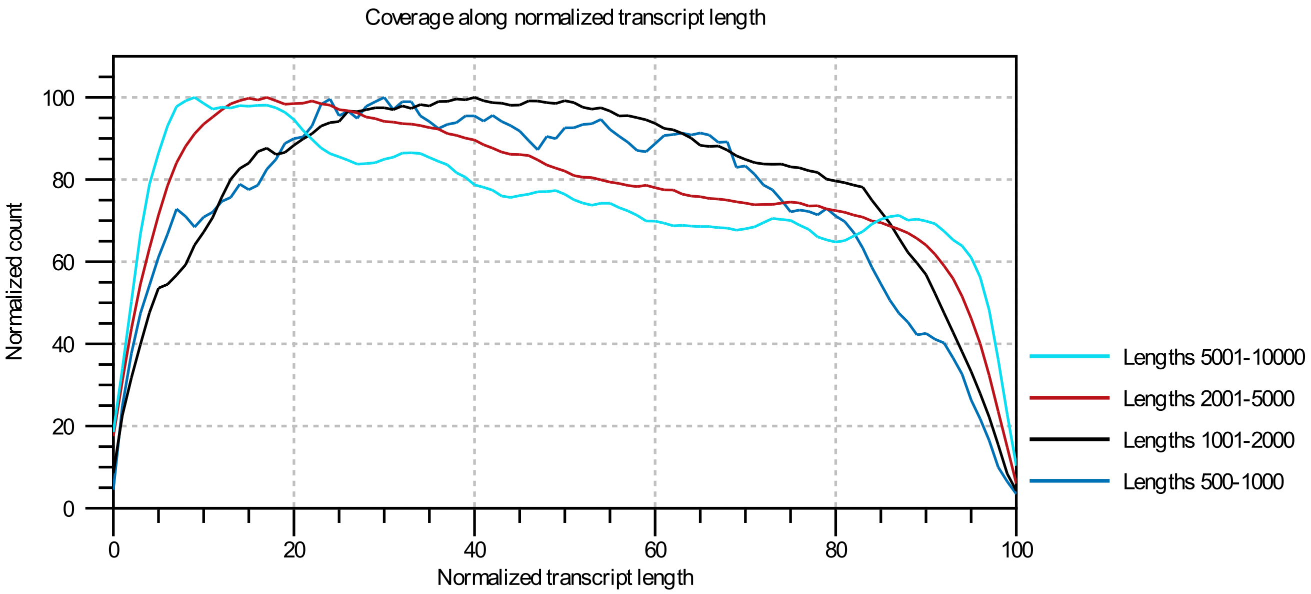


| | |
|---|---|
| Expected coverage bias | Unbiased |
| Difference between average 3’ and 5’ normalized counts | -16.27 |
| % reads mapping to transcripts > 10000 bp | 3.20 |
| % reads mapping to transcripts 5001 - 10000 bp | 16.76 |
| % reads mapping to transcripts 2001 - 5000 bp | 43.48 |
| % reads mapping to transcripts 1001 - 2000 bp | 25.61 |
| % reads mapping to transcripts 500 - 1000 bp | 10.20 |
| % reads mapping to transcripts < 500 bp | 0.76 |

The plot shows the normalized coverage across a transcript body for four different groupings of transcript length. The lines should be flat in the center of the plot, and the plot should be approximately symmetric. An erratic line may indicate that there are few transcripts in the given length range.
